# Clinical findings of West Nile virus infection in hospitalized patients, New York and New Jersey, 2000.

**DOI:** 10.3201/eid0704.010409

**Published:** 2001

**Authors:** D Weiss, D Carr, J Kellachan, C Tan, M Phillips, E Bresnitz, M Layton, West Nile Virus Outbreak Response Working Group

**Affiliations:** New York City Department of Health, 125 Worth St., New York, NY 10013, USA. Dweiss@health.nyc.gov

## Abstract

Outbreaks of West Nile (WN) virus occurred in the New York metropolitan area in 1999 and 2000. Nineteen patients diagnosed with WN infection were hospitalized in New York and New Jersey in 2000 and were included in this review. Eleven patients had encephalitis or meningoencephalitis, and eight had meningitis alone. Ages of patients ranged from 36 to 87 years (median 63 years). Fever and neurologic and gastrointestinal symptoms predominated. Severe muscle weakness on neurologic examination was found in three patients. Age was associated with disease severity. Hospitalized cases and deaths were lower in 2000 than in 1999, although the case-fatality rate was unchanged. Clinicians in the Northeast should maintain a high level of suspicion during the summer when evaluating older patients with febrile illnesses and neurologic symptoms, especially if associated with gastrointestinal complaints or muscle weakness.


### Clinical Findings of West Nile Virus Infection in Hospitalized Patients, New York and New Jersey, 2000

**Don Weiss,* Darcy Carr,* Jacqueline Kellachan,* Christina Tan,†‡ Michael Phillips,*‡ Eddy Bresnitz,† and Marcelle Layton,* for the West Nile Virus Outbreak Response Working Group**

*New York City Department of Health, New York, New York, USA; †New Jersey Department of Health and Senior Services, Trenton, New Jersey, USA; ‡Centers for Disease Control and Prevention, Atlanta, Georgia, USA[1]

Outbreaks of West Nile (WN) virus occurred in the New York metropolitan area in 1999 and 2000. Nineteen patients diagnosed with WN infection were hospitalized in New York and New Jersey in 2000 and were included in this review. Eleven patients had encephalitis or meningoencephalitis, and eight had meningitis alone. Ages of patients ranged from 36 to 87 years (median 63 years). Fever and neurologic and gastrointestinal symptoms predominated. Severe muscle weakness on neurologic examination was found in three patients. Age was associated with disease severity. Hospitalized cases and deaths were lower in 2000 than in 1999, although the case-fatality rate was unchanged. Clinicians in the Northeast should maintain a high level of suspicion during the summer when evaluating older patients with febrile illnesses and neurologic symptoms, especially if associated with gastrointestinal complaints or muscle weakness.

Following the emergence of West Nile (WN) virus in New York in 1999, state and local health departments in the eastern United States, in conjunction with the Centers for Disease Control and Prevention (CDC), established surveillance systems for detecting WN virus activity (1). New York City and New Jersey established active and enhanced passive surveillance systems for human disease that encouraged physician, infection control practitioner, and laboratory reporting of suspected cases and provided testing for WN virus. This report details the clinical characteristics of 19 hospitalized human cases that occurred during the summer and fall of 2000; all patients resided in either New York City (NYC) or New Jersey.

### Methods

Enhanced surveillance for WN virus human disease in New York and New Jersey during 2000 was instituted to facilitate timely reporting of viral meningoencephalitis and to ensure rapid and accurate laboratory testing for WN virus. In NYC, encephalitis and viral meningitis are reportable conditions. From May to October 2000, the following measures were implemented by the NYC Department of Health to supplement existing passive surveillance: 1) Enhanced passive surveillance—To encourage physician reporting citywide, information on WN virus reporting and testing procedures was widely disseminated to the medical community through invited presentations by departmental medical staff, an agency publication mailed to >65,000 health-care providers, and biweekly broadcast facsimile and e-mail alerts to all NYC hospitals; 2) Hospital-based active physician surveillance—Neurologists, infectious disease consultants, intensive-care physicians, and chief medical residents at 18 sentinel NYC hospitals were called every 2 weeks to ascertain potential cases meeting clinical criteria; 3) Hospital-based active laboratory surveillance—Laboratories at 12 sentinel NYC hospitals submitted all cerebrospinal fluid (CSF) specimens suggestive of a viral cause for WN virus testing at the NYC health department (CSF with negative Gram stain and culture with either a CSF leukocyte count $\geq 5/mm^3$ or protein $\geq$40 mg/dL). A special unit was established within the Communicable Disease Program of the NYC Department of Health to implement these additional surveillance activities. This unit ensured that all suspected cases were prioritized for next-business-day specimen collection and transportation to the city's Public Health Laboratories for WN virus testing.

In New Jersey, bacterial meningitis and encephalitis are reportable to the New Jersey Department of Health and Senior Services (NJDHSS). Active, hospital-based surveillance by infection control practitioners targeted patients admitted with the diagnoses of aseptic meningitis or encephalitis in 42 hospitals in six northern counties. Passive surveillance was enhanced through the distribution of reporting protocols, surveillance criteria, and WN virus educational materials to the medical community. Medical

Address for correspondence: Don Weiss, New York City Department of Health, 125 Worth St., Box 22A, New York, NY 10013, USA; fax: 212-676-6091; e-mail: Dweiss@health.nyc.gov

[1]A. Labowitz, J.A. Greenko, B. Maldin, B. Edwin, I. Poshni, A. Fine, New York City Department of Health; R. Lanciotti, Centers for Disease Control and Prevention; F. Sorhage, C. Farello, D. Adam, B. Wolf, New Jersey Department of Health and Senior Services; A Dupius, L. Kramer, New York State Department of Health.

providers were reminded to notify the NJDHSS of suspected cases. NJDHSS conducted weekly follow-up with physicians and infection control practitioners to review the status of pending cases.

A patient was considered to have a confirmed WN case if he or she was hospitalized with an illness associated with neurologic manifestations consistent with meningitis or encephalitis, and had laboratory confirmation of WN infection. The four laboratory confirmation criteria used for WN infection, established by CDC (1), are as follows: 1) isolation of WN virus from, or demonstration of WN viral antigen or genomic sequences in tissue, blood, CSF, or other body fluid; 2) demonstration of immunoglobulin M (IgM) antibody to WN virus in CSF by IgM-capture enzyme immunoassay (EIA); 3) ≥4-fold serial change in plaque-reduction neutralizing antibody titer (PRNT) to WN virus in paired, appropriately timed serum or CSF samples; and 4) demonstration of both WN virus-specific IgM (by EIA) and IgG (screened by EIA and confirmed by PRNT) antibody in a single serum specimen.

Patients were classified into three clinical categories: meningitis, if they had fever plus headache, stiff neck, or photophobia; encephalitis, if they had altered mental status or other cortical signs; or meningoencephalitis, if they met both criteria. The categories of encephalitis and meningoencephalitis were combined as "any encephalitis" in some analyses. All syndromes required abnormal CSF findings consistent with a viral cause (CSF with negative Gram stain and culture with either a CSF leukocyte count ≥5/mm$^3$ or protein ≥40 mg/dL).

IgM-capture EIA was performed at either the NYC Public Health Laboratories or the New Jersey Public Health and Environmental Laboratory; confirmation of positive results by PRNT was performed by CDC or the NY State Department of Health. Viral neutralization testing followed CDC protocol (R. Lanciotti, pers. commun.). Real-time Taqman polymerase chain reaction (PCR) was performed at CDC. Medical chart reviews and patient interviews were completed on all patients with positive tests for WN virus by IgM-capture EIA.

Supplementary medical chart reviews by the physician authors were completed in November-December 2000 after confirmation of initial results. Information abstracted included demographics, symptoms, chronology of illness, admission diagnosis, clinical findings, coexisting illness, laboratory findings, hospital course, diagnostic procedures, complications, level of neurologic involvement, discharge condition, and diagnoses. If a symptom was not specifically mentioned or a physical finding was not noted in the medical record, it was considered to be absent. Patient addresses were geocoded and mapped to compare the geography of the 2000 and 1999 epidemics. Descriptive statistics and Fisher's exact p values were calculated with SPSS (SPSS Chicago, version 10.0) and Epi Info (CDC, Atlanta, version 6.04b). Mapping was done in ArcView (ERSI, Redlands, CA, version 3.2).

### Results

### Demographics

Nineteen hospitalized WN virus patients were identified in 2000, 14 (74%) from New York and 5 (26%) from New Jersey. The 14 New York cases were from four of the five NYC counties; 10 were from Richmond County (Staten Island), 2 from Kings County (Brooklyn), and one each from New York County (Manhattan) and Queens County. The New Jersey cases occurred in Hudson County (2 cases) and in Bergen, Morris, and Passaic counties (1 case each). Eleven (58%) were male and eight (42%) were female. The median age was 63 years (range 36-87). Eight patients (42%) were ≥65 years of age, and six (32%) of these were ≥75 years of age.

### Clinical Illness and Hospital Course

Nine patients were classified as having encephalitis, eight with meningitis and two with meningoencephalitis. All eight patients ≥65 years of age had either encephalitis or meningoencephalitis, accounting for 73% of all cases with encephalitis. The mean age of patients with encephalitis was 71 years (standard deviation [SD]=11.7), compared with 51 years (SD=14.5) for patients with meningitis alone. A history of hypertension (as documented in the past medical history section of the medical record) was present in 8 (73%) of 11 patients with either encephalitis or meningoencephalitis, compared with 3 (38%) of 8 patients with meningitis alone.

The median and mean time periods from symptom onset to hospitalization were 3 and 7.7 days, respectively (range 0-48). One patient became symptomatic 3 days after being hospitalized for an unrelated, noninfectious condition, and another was hospitalized 48 days after the initial onset of symptoms. Patients' onset dates occurred within a 9-week interval from July 19 to September 12, 2000 (Figure 1). The median length of hospital stay was 7 days (range 1-72), and patients ≥65 years of age had a longer median length of stay than those <65 years (11 days vs. 6 days). Five patients were admitted to intensive care units (ICU), and two required mechanical ventilation. The median length of stay in the ICU was 17 days (range 2-47 days).

The patient's temperature on admission ranged from 36.6°C to 40.6°C (median 38.6°C), and 14 patients were febrile upon arrival at the emergency department (fever defined as temperature of ≥38.0°C). Three patients became febrile during their hospital stay, and two did not have documented fever and were determined to have WN infection based on a clinical diagnosis of meningitis or encephalitis and laboratory confirmation. The mean duration of fever was 2.9

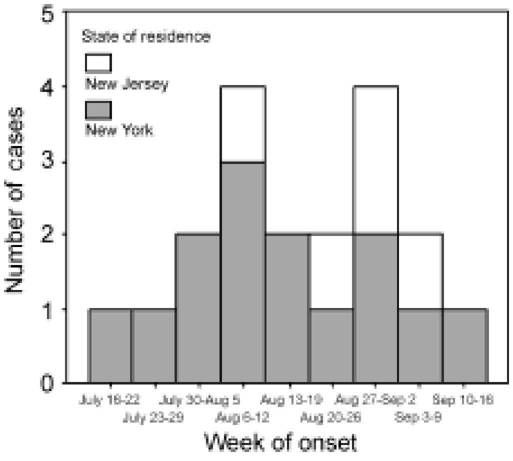

Figure 1. New York City metropolitan area West Nile virus epidemic curve, 2000.

days (range 0-6 days). The two patients without fever were <41 years old. One denied a history of fever, while the other was admitted 7 weeks after onset with an unclear history of fever.

The frequency of symptoms and clinical findings is presented in Table 1. Neurologic and gastrointestinal findings predominated. Of the 19 cases, 16 (84%) presented with at least one neurologic complaint (headache, stiff neck, photophobia, muscle weakness, or change in mental status). Seventeen patients (89%) had one or more abnormalities on neurologic examination. Motor exams in three patients demonstrated muscle weakness (strength <5/5); of the six with abnormal reflexes, four were hyporeflexive, and two had abnormal plantar responses; the two patients with cerebellar abnormalities were ataxic. Both patients with cranial nerve abnormalities died; one had nystagmus and the other had a depressed gag reflex.

Table 1. Frequency of clinical findings in West Nile virus patients, New York and New Jersey, 1999 and 2000

| Symptom or physical finding | Frequency No. (%) |
|---|---|
| Fever | 17 (90) |
| Fatigue | 12 (63) |
| Altered mental status | 11 (58) |
| Headache | 11 (58) |
| Reported weakness | 8 (42) |
| Nausea | 8 (42) |
| Vomiting | 8 (42) |
| Myalgia | 6 (32) |
| Photophobia | 6 (32) |
| Abnormal reflexes | 6 (32) |
| Stiff neck | 6 (32) |
| Abdominal pain | 4 (21) |
| Motor weakness | 3 (16) |
| Cough | 3 (16) |
| Diarrhea | 3 (16) |
| Seizures | 3 (16) |
| Arthralgia | 2 (11) |
| Cerebellar abnormality | 2 (11) |
| Cranial nerve palsy | 2 (11) |
| Shortness of breath | 2 (11) |

Eleven patients (58%) had at least one gastrointestinal symptom or had abnormal abdominal findings. Three patients had rash. In two of these, the rash was truncal and either macular or papular; the rash in the third patient was not described.

Seventeen patients initially received antibiotics, and eight were treated with acyclovir for presumptive herpes encephalitis. One patient was comatose and was treated with oral ribavirin and alpha-interferon without improvement. This patient died of complications 16 weeks after transfer to a long-term care facility.

### Laboratory and Radiology Findings

Eighteen cases were diagnosed based on positive CSF IgM-capture EIA; 9 of these were confirmed by a fourfold rise in PRNT antibodies. An appropriately timed acute- or convalescent-phase serum sample could not be obtained for the remaining patients. Thirteen patients also had real-time Taqman PCR testing of CSF, and one was positive (obtained 8 days after illness onset). One other patient, a 43-year-old man, did not have sufficient CSF for testing; his case was confirmed by positive serum IgM-capture EIA and PRNT results in a single serum specimen. Seventeen patients had an initial CSF pleocytosis, and 15 of these had a differential cell count performed. Nine patients had a predominance of neutrophils (≥50%) in the CSF (Table 2). The presence of neutrophilic pleocytosis was not associated with the more severe presentation of encephalitis (p=0.5).

Three patients had hemoglobin values >2 SD below the gender-specific mean values (73-year-old man, a 73-year-old woman, and an 87-year-old woman). A low platelet count (<150,000/mm$^3$) occurred in one patient with a previous history of thrombocytopenia, and another patient had a low total leukocyte count (4,400/mm$^3$). Abnormal serum sodium levels (Na <135 mmol/L) occurred in eight (42%) patients. This finding was noted more frequently in those with encephalitis (hyponatremia in 6/11 with any encephalitis vs. 2/8 with meningitis). In two patients with hyponatremia and encephalitis, the low serum sodium could be explained by

Table 2. West Nile patient laboratory findings, New York and New Jersey, 1999 and 2000

| Test | Number tested (%) | Mean value or N with condition (range) | Normal values (2) |
|---|---|---|---|
| CSF | | | |
| Leukocyte count, mean | 19 (100) | 308 mm$^3$ (0-1782) | 0-5 cells/mm$^3$ |
| Red cell count, mean | 16 (84) | 115/mm$^3$ (0-700) | 0 cells/mm$^3$ |
| Protein, mean | 19 (100) | 111 mg/dL (56-555) | 15-50 mg/dL |
| Glucose, mean | 19 (100) | 67 mg/dL (48-95) | 50-80 mg/dL |
| Differential,[a] ≥50% neutrophils | 15 (79) | 9 (1-100%) | All mononuclear cells |
| Complete blood cell count | | | |
| Leukocyte count, mean | 19 (100) | 10,600/mm$^3$ (4,400-19,700) | 4,500-11,000/mm$^3$ |
| Differential cell count,[a] >77% segs + bands | 18 (95) | 11 (55-96%) | 59% ± 18 |
| Hemoglobin (male), mean | 11 (100) | 14.5 g/dL (11.8-16.5) | 15.5 g/dL ± 1.1 |
| Hemoglobin (female), mean | 8 (100) | 12.7 g/dL (10.5-14.6) | 13.7 g/dL ± 1.0 |
| Other laboratory | | | |
| Hyponatremia, serum Na <135 mmol/L | 19 (100) | 8 (42%) | 135-145 mmol/L |
| Elevated AST, >twice upper limit | 17 (90) | 4 (24%) | 10-35 units/L |
| Elevated ALT, >twice upper limit | 15 (79) | 1 (7%) | 20-48 units/L |
| Elevated total bilirubin, >twice upper limit | 16 (84) | 3 (19%) | 0.3-1.0 mg/dL |

[a]Values are the number of patients with the laboratory finding; ranges are the values of all patients.
CSF = cerebrospinal fluid; AST = aspartate aminotransferase; ALT = alanine aminotransferase; segs = segmented neutrophils.

other causes. One had spurious hyponatremia caused by hyperglycemia (glucose=598 mg/dL), and the other had elevated blood urea nitrogen (34 mg/dL) and urine specific gravity (1.025) consistent with dehydration.

Radiographic imaging of the brain was conducted in 18 patients (95%). Eleven had computerized tomography, two had magnetic resonance imaging, and five had both. Abnormalities were noted in 10 (56%) patients. Eight had nonacute abnormalities with either evidence of an old infarct, mild communicating hydrocephalus, atrophy, leukomalacia, or ischemia. Two had acute inflammatory changes: one had leptomeningeal enhancement and the other had periventricular hyperintensity of the white matter. Seven of the eight patients with evidence of old brain injury had encephalitis, compared with two of eight with normal brain imaging studies. A patient with Parkinson's disease, diabetes, and hypertension was the only patient in the series to have an electromyogram. Results showed moderate-to-severe peripheral neuropathy, mainly demyelinating, with involvement of sensory and motor neurons consistent with Guillain-Barré syndrome.

### Outcome

As recorded in discharge summaries, 10 patients (53%) recovered but not to their functional level before illness, 7 (37%) recovered fully, and 2 died (11%). Both deaths occurred in patients >80 years of age, and neither had an autopsy. Thirteen (68%) patients were discharged to home, 4 (21%) were discharged to a long-term care facility, one (5%) died in the hospital, and the location of discharge of one patient (5%) was unknown. When discharged from the acute-care facility, seven (37%) were fully ambulatory, five (26%) were ambulatory with assistance, two (11%) were bedridden, and the condition was unknown for four (21%) patients. Five (26%) patients required in-hospital physical therapy or consultation, three (16%) required speech therapy or consultation, and two (11%) had occupational therapy or consultation.

### Temporal and Geographic Trends

WN virus patients had onset dates in the 9-week period from July 19 to September 12, 2000. The temporal distribution of cases was bimodal, with four cases occurring during the weeks of August 6-12 and August 27-September 2 (Figure 1). The epicenter was in Staten Island with the hospitalized human cases encompassing an area of 1,520 square miles (Figure 2). During the first 6 weeks of the epidemic, nine cases occurred on Staten Island, one in Brooklyn, and two in New Jersey. In the final 3 weeks of the epidemic, one case occurred on Staten Island, three cases occurred in other New York City boroughs (one each in Brooklyn, Manhattan, and Queens), and three cases occurred in New Jersey.

### Discussion

Nineteen hospitalized adults were diagnosed with WN virus infection in the New York metropolitan area in 2000, a decline of 68% from 1999. The epicenter was located in Staten Island, approximately 20 miles west and south of the 1999 epicenter in northern Queens. Most patients had a febrile illness associated with meningeal signs, altered mental status, or both. The median age of hospitalized patients was lower in 2000 (63 vs. 71 years), and the proportion with encephalitis decreased from 63% to 58% (p=0.1). Gastrointestinal complaints were common, and severe motor weakness was reported less frequently than in 1999 (16% in 2000 vs. 27% in 1999). In 1999, seven deaths were caused by WN virus; in 2000 two were. The case-fatality rates for the 2 years do not differ statistically (11.9% in 1999, 10.5% in 2000, p=0.6).

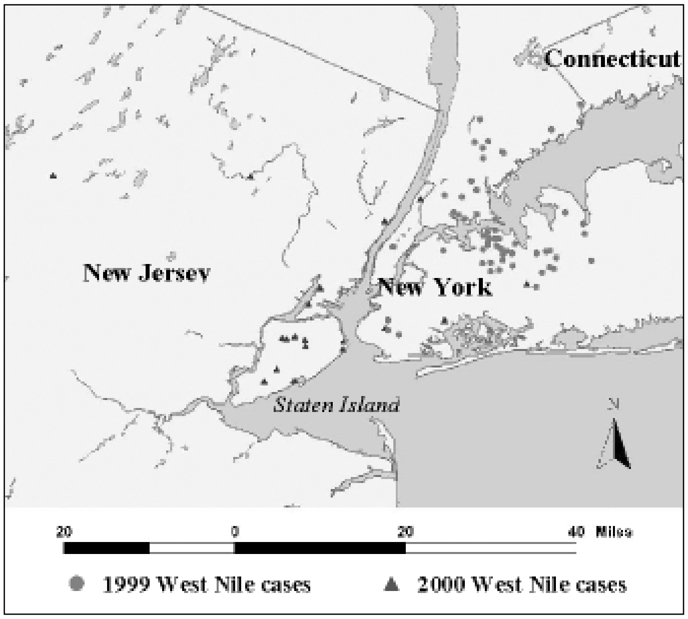

Figure 2. Metropolitan New York area hospitalized West Nile virus patients, 1999-2000.

Routine laboratory findings were nonspecific. CSF findings were consistent with a nonbacterial inflammatory process. Mild hyponatremia was found in eight patients. The syndrome of inappropriate secretion of antidiuretic hormone has been described in viral meningitis, St. Louis encephalitis, and WN virus (3-5). Two of eight patients with hyponatremia had other reasons for this finding (one with suspected dehydration and the other with hyperglycemia), and information on the use of antihypertensive medications, including diuretics, was not collected nor were urine osmolalities measured. A possible association of WN infection with this syndrome cannot be determined from this case series and requires further investigation.

Reasons for the differences seen in the number of human cases over the two epidemic years are speculative. Aggressive mosquito and larval control activities, particularly on Staten Island, may have reduced the infected mosquito population enough to diminish WN virus transmission to humans. Increased immunity in the resident avian population may have also prevented the re-establishment of an enzootic amplification cycle sufficient to cause significant human disease in Queens, the epicenter of the 1999 outbreak. Evidence from an avian serosurvey conducted after the 1999 epidemic supports this theory, since 51% of birds captured in Queens and 2% of those in Staten Island tested positive for WN virus (6).

WN virus infection in hospitalized cases in 2000 occurred over a 9-week period from mid-July to mid-September. A greater proportion of cases occurring outside Staten Island were recognized toward the end of this interval, which may relate to differences in vector ecology or control measures used. The timing of the 2000 epidemic curve closely resembles that of the recent outbreak in Romania (7) and preceded the 1999 New York epidemic curve by 2 weeks. The earlier onset in 2000 may have resulted from enhanced surveillance efforts that were not in place before the 1999 outbreak was recognized. In the 1996 Romanian outbreak, the predilection for WN virus to cause severe disease with increasing age and the frequency of gastrointestinal complaints were similar to findings in this series. Most encephalitis cases were in persons >50 years of age; vomiting occurred in 63% and diarrhea in 12% of cases (8). The propensity of WN virus to affect the elderly more seriously has been seen with other flaviviruses, most notably St. Louis encephalitis (9). The common contributing factors of age, hypertension, and previous brain insult may relate to a decline in the integrity of the blood brain barrier and facilitated access of WN virus to the central nervous system.

The interpretation of the findings of this case series is limited because of the small number of cases. Only hospitalized patients were included, and most WN virus infections are subclinical. Two additional nonhospitalized WN fever cases, one in Connecticut and one in New Jersey, were detected through surveillance and were not included in this case series (10). Focusing on the most severely ill obscures the true spectrum of WN illness. A 1999 serosurvey in Queens, New York, estimates that for every hospitalized case of WN virus infection there were 24 mild febrile and 110 subclinical illnesses (F. Mostashari, pers. commun.). Surveillance in 2000 focused on adults with aseptic meningitis or encephalitis; patients <18 years old were only included if they had encephalitis. The active laboratory surveillance component, however, included patients <18 years old. Four hundred fifty-three CSF specimens were received through active laboratory surveillance; 13 (3%) were from children <18 years. No positive results in children were found.

Another limitation was that the data described were abstracted from medical records that varied greatly in their completeness and legibility. The frequency of missing, missed, and omitted information was approximately 5%-10%. For some analyses, clinical information not located in the medical record was coded as negative, possibly introducing bias that could have produced spurious or hidden real associations.

WN virus appears to have established an enzootic cycle in the northeast United States with positive avian or mosquito findings extending from New Hampshire to North Carolina (11). Clinicians practicing along the eastern seaboard should consider this diagnosis when evaluating febrile patients during the summer months with neurologic complaints, especially those with a gastrointestinal prodrome or muscle weakness.

### Acknowledgments

We thank the medical staff, record departments, and laboratories of New York City and New Jersey State hospitals; D. Minucci, J. Kessler, M. Shah, K. Levin, J. Lapadula, D. Das, and F. Mostashari.

Dr. Weiss is the medical director of the Surveillance Unit, Communicable Disease Program, New York City Department of Health. His interests include the epidemiology of emerging and reemerging infectious diseases, particularly as they occur in the urban environment.

### References

1. Centers for Disease Control and Prevention. National West Nile Virus Surveillance System, 2000: Final Plan, May 2000, Atlanta, GA. [document online] Available from url: http://www.cdc.ncidod/dvbid/westnile/resources/WN_Final_Plan_2000_05_26_31.pdf
2. Jacobs DS, DeMott WR, Grady HJ, Horvat RT, Huestis DW, Kasten BL. Laboratory test handbook. Cleveland: Lexi-comp, Inc; 1996.
3. Fajardo JE, Stafford EM, Bass JW, Roscelli JD, Sato AK, Claybaugh. Inappropriate antidiuretic hormone in children with viral meningitis. Pediatr Neurol 1989;5:37-40.
4. White MG, Carter NW, Rector FC, Seldin DW, Drewey SJ, Sanford JP, et al. Pathophysiology of epidemic St. Louis encephalitis. Inappropriate secretion of antidiuretic hormone. Ann Intern Med 1969;71:691-702.
5. Cernescu C, Ruta SM, Tardei G, Grancea C, Moldoveanu L, Spulbar E, et al. A high number of severe neurologic clinical forms during an epidemic of West Nile infection. Rom J Virol 1997;48:13-25.
6. Komar N, Panella NA, Burns J, Disza S, Mascarenhas TM, Talbot TO. Serologic evidence for West Nile virus infection in birds in the New York City vicinity during an outbreak in 1999. Emerg Infect Dis 2001;7:621-5.
7. Tsai TF, Popovici F, Cernescu C, Campbell GI, Nedeclu I, for the Investigative Team. West Nile encephalitis epidemic in southern Romania. Lancet 1998;352:767-71.
8. Ceausu EM, Erscoiu S, Calistru P, Ispar D, Doroboat O, Homos M. Clinical manifestations in West Nile virus outbreak. Rom J Virol 1997;48:3-11.
9. Marfin AA, Bleed DM, Lofgren JP, Olin AC, Savage HM, Smith GC, et al. Epidemiologic aspects of a St. Louis encephalitis epidemic in Jefferson County Arkansas, 1991. Am J Trop Med Hyg 1993;49:30-7.
10. Centers for Disease Control and Prevention. Serosurveys for West Nile virus infection—New York and Connecticut counties, 2000. MMWR Morb Mortal Wkly Rep 2001;50:37-9.
11. Centers for Disease Control and Prevention. Update: West Nile virus activity—Eastern United States, 2000. MMWR Morb Mortal Wkly Rep 2001;49:1044-7.
